# Cutaneous Scarring: A Clinical Review

**DOI:** 10.1155/2009/625376

**Published:** 2010-02-10

**Authors:** Richard Baker, Fulvio Urso-Baiarda, Claire Linge, Adriaan Grobbelaar

**Affiliations:** ^1^Queen Victoria Hospital NHS Foundation Trust, Holtye Road, East Grinstead, West Sussex RH19 3DZ, UK; ^2^Department of Plastic Surgery, Bradford Royal Infirmary, Duckworth Lane Bradford, West Yorkshire BD9 6RJ, UK; ^3^RAFT Institute of Plastic Surgery, The Leopold Muller Building, Mount Vernon Hospital, Rickmansworth Road, Northwood, Middlesex HA6 2RN, UK; ^4^Department of Plastic Surgery, Royal Free Hospital Hampstead NHS Trust, Pond Street, London NW3 2QG, UK

## Abstract

Cutaneous scarring can cause patients symptoms ranging from the psychological to physical pain. Although the process of normal scarring is well described the ultimate cause of pathological scarring remains unknown. Similarly, exactly how early gestation fetuses can heal scarlessly remains unsolved. These questions are crucial in the search for a preventative or curative antiscarring agent. Such a discovery would be of enormous medical and commercial importance, not least because it may have application in other tissues. In the clinical context the assessment of scars is becoming more sophisticated and new physical, medical and surgical therapies are being introduced. This review aims to summarise some of the recent developments in scarring research for non-specialists and specialists alike.

## 1. Introduction

Cutaneous scarring is inevitable following damage to more than 33.1% of the thickness of the skin either through trauma or surgery [1]. An estimated 23 million people in the UK have significant scars and although most of these are asymptomatic a proportion cause psychological and physical morbidity whilst some are pathological [2]. Although scars are permanent there are established methods of improving symptomatic scars medically or with surgical revision [3]. However, the evidence base for most of these treatments is poor and their efficacies are limited [3].

An effective cutaneous antiscarring agent would have profound benefits in relation to trauma and burns but in addition may have efficacy in the prevention of postsurgical abdominal adhesions and in the treatment of medical fibroses such as renal, pulmonary, and hepatic. This paper aims to update doctors of all specialties on the current state of the art regarding both research and treatment of cutaneous scarring.

## 2. Methods

Pubmed and Medline were searched using terms “scar” and “cutaneous” from 1998 onwards. Personal archives were also consulted. The articles selected comprise original papers, reviews, recommendations, and consensus reports. Whilst some of the studies are prospective randomized controlled trials, many are prospective or retrospective observational reports or laboratory based studies. Although the evidence for many antiscarring therapies is at this stage weak or in the preclinical or development phases, much of this research is referenced for completeness. 

### 2.1. How Do Scars Form?

**Figure 1:**
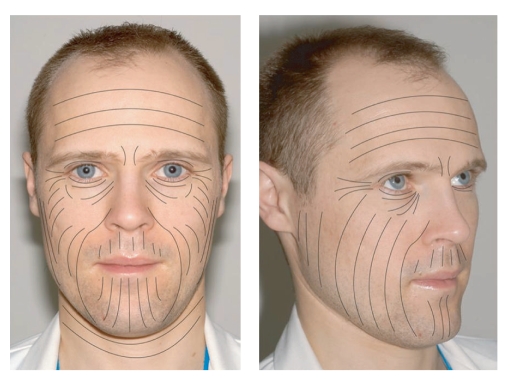
The lines of Langer or relaxed skin tension lines.

A cutaneous scar is defined as dermal fibrous replacement tissue and results from a wound that has healed by resolution rather than regeneration [4]. Final appearance is largely influenced by the interval between wounding and complete healing 2 to 3 weeks later. It is here that the doctor can do most to prevent the development of pathological scarring. Incisions should be placed within or parallel to the lines of Langer (wrinkle lines) (Figure 1) and away from anatomical sites prone to pathological scarring such as shoulders, sternum, across joints, or near orifices. Wounds should be closed with the minimum possible tension and paper tape applied to redistribute the tension over a greater surface area. Infection, foreign bodies (e.g., retained sutures) or prolonged healing (beyond 2 weeks) will all contribute to poorer scarring [5]. 

Once the scar has formed it undergoes several distinct macro- and microscopic changes during the maturation process and is complete on average after 1 year [6]. Patients under 30 years exhibit a slower rate of scar maturation and poorer final appearance than patients over 55 years [6]. The redness of a scar fades after 7 months and in contrast with rubor elsewhere does not reflect an inflammatory process (after the first month) [7]. The scar is devoid of dermal appendages and never reaches the same tensile strength as the surrounding skin [8]. 

**Figure 2:**
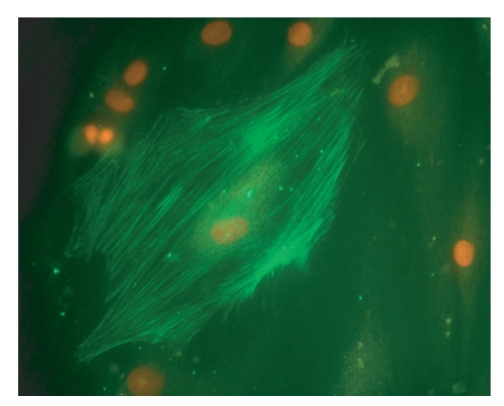
A human myofibroblast, ×40 magnification. The nucleus is stained orange with propidium iodide and the filaments of *α*-smooth muscle actin are immunostained green.

Scar tissue consists mainly of disorganised collagenous extracellular matrix. This is produced by myofibroblasts (Figure 2) which differentiate from dermal fibroblasts in response to wounding which causes a rise in the local concentration of transforming growth factor-*β* (TGF-*β*). TGF-*β* is an important cytokine associated with fibrosis in many tissue types [8]. Myofibroblasts are characterised by contractile microfilaments of smooth muscle proteins such as *α*-smooth muscle actin, which give scar tissue its contracting property and together with TGF-*β* are the principal targets of attempts to suppress scarring [9, 10].

### 2.2. How Are Scars Assessed?

**Figure 3:**
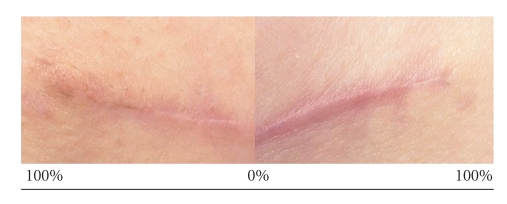
The global scar comparison scale. The photographic records of each scar are placed side by side over a double-ended visual analogue scale which represents percentage scar improvement. The 0% rating equates to an assessors opinion that there is no detectible difference between the two scars; whereas the 100% rating on either side means that that particular scar is so improved that it is imperceptible from the surrounding normal skin.

Histopathological examination is the gold standard for scar assessment but is not appropriate for monitoring the response to therapy of scars in a clinic setting or in the context of clinical trials, so clinical tools have been developed that facilitate objective assessment of scars. The first such scale, the Vancouver Burn Scar Assessment Scale rates scars on pigmentation, vascularity, pliability, and height [11]. This initial concept has been developed to make descriptions numerical, to include scar location, patient observation and to broaden application to linear nonburn scars [4, 12, 13]. To compensate for the high intrapatient variation in scars the Global Scar Comparison Scale has been recently designed and validated by Renovo Ltd specifically for assessing new antiscarring therapies [14]. Images of treated and placebo-treated scars are compared side by side combining a ranking and visual analogue-scale measurement in one assessment allowing detection of smaller differences in scarring outcome (Figure 3). However, all these scales are inevitably limited by their subjectivity so several instruments have been designed to circumvent this problem by objectively measuring certain properties of a scar. These include redness/erythema (e.g., Minolta Chromameter), pigmentation (e.g., DermaSpectometer), thickness (ultrasound), surface area and texture (digital photography and optical or mechanical profilometers), and suppleness (e.g., Cutometer) [10, 13, 15, 16]. Finally, there are experimental 3D imaging technologies that can accurately calculate scar volume and digital image analysis is also likely to become more important [17].

### 2.3. Why Do Early-Gestation Fetuses Not Scar?

The possibility of manipulating scar formation scarless healing was raised by the observation of scarless healing of amputation stumps caused by amniotic bands in a 20-week old human fetus [18]. Although the nonscarring fetus is generating new skin and is bathed in amniotic fluid, these conditions remain in late gestation when the fetus does scar. However, there are three important differences in the early gestation fetus that may explain the different response to wounding.

**(1) Reduced Inflammatory Response in the Early Gestation Fetus [19]**

Fetal skin has fewer macrophages and lymphocytes compared to adult skin and inflammatory cells persist for less time in the wound [19–21]. This maybe due to the reduced degranulation of fetal platelets, their lower PDGF and TGF-*β*1 and 2 content, or reduced aggregation [22–25].

**(2) Differing Growth Factor Profile [19]**

Levels of the profibrotic TGF-*β*1 and TGF-*β*2 are higher in scarring fetal rat wounds than in non-scarring fetal wounds; whereas levels of the antifibrotic TGF-*β*3 are higher in non-scarring wounds and this is repeated in human fetal skin [26, 27]. Other cytokines are implicated such as VEGF (antifibrotic), PDGF, and FGF-2 and non-cytokines such as hydrogen peroxide (profibrotic) [15, 28, 29].

**(3) Innately Different Fibroblasts [30]**

Fetal fibroblasts migrate faster, show less propensity to differentiate into myofibroblasts and respond differently to certain scarring-associated cytokines such as insulin-like growth factors and TGF-*β*1 than their adult counterparts [31–34]. Although TGF-*β*1 autostimulates expression of its own gene in scarring fibroblasts, non-scarring fetal fibroblasts display only a short-lived response to TGF-*β*1 [35].

### 2.4. What Are Pathological Scars?

**Figure 4:**
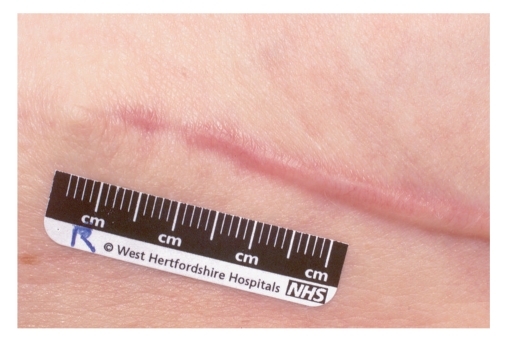
A hypertrophic postsurgical scar.

**Figure 5:**
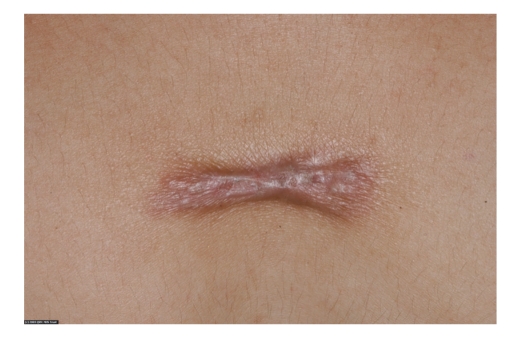
Presternal keloid scar.

Hypertrophic (Figure 4) and keloid scars (Figure 5) are both forms of excessive dermal fibrosis. They are both characterised by increased vascularity, high mesenchymal density, inflammatory cell infiltration, and a thickened epidermis [36]. However, their clinical characteristics and pathologies intrinsically differ with keloids being the more complex, extreme, and challenging to treat (see Table 1). The treatment of both of these types of scar can be protracted and is best managed by a specialist from the outset.

### 2.5. Why Do Some Scars Become Pathological?

Pathological scars are thought to be caused by disordered regulation of wound cellularity and collagen synthesis [37]. Growth factors, extracellular matrix components, abnormal collagen turnover, sebum immunoreactivity, genetic influences, and tension have all been implicated [38]. Pathological scars are hyperresponsive to TGF-*β*1 with connective tissue growth factor expression increases 150-fold and 100-fold in hypertrophic and keloid scars, respectively, in response to TGF-*β*1 compared with normal fibroblasts [39]. Failure of apoptosis may also have a role, keloid fibroblasts in particular are highly resistant to fatty acid synthase-mediated apoptosis and the tumour suppressor genes, p53 and p63, which are involved in the induction of apoptosis, are upregulated [40–43]. There appear to be predisposing systemic traits too. Burn patients who subsequently develop hypertrophic scars have higher IL-10, TGF-*β* serum levels, and elevated numbers of IL-4-positive Th 2 cells early after burn injury compared with those that develop normal scars [44]. Familial clustering and the markedly higher predisposition of patients of Afro-Carribean origin to developing keloids suggest that there is a major genetic contribution with keloid susceptibility loci having been found on chromosomes 2q23 and 7p11 [45, 46].

### 2.6. Why Do We Need Antiscarring Therapies?

As problematic scars are often caused by an intrinsic dysfunction of the process of wound healing, surgery simply serves to recreate the precipitating event and commonly results in recurrence. Several physical, medical, and surgical therapies have therefore been developed to both prevent and treat poor scarring.

### 2.7. What Physical Antiscarring Therapies Exist?

Silicone gel sheeting remains first-line treatment for normal and hypertrophic scars and has been proved efficacious in a large metanalysis [47, 48]. The mechanism of action remains unclear. Pressure therapy in the form of compression garments and hydrotherapy are widely used particularly for hypertrophic burn scars; however strong evidence for their efficacy is lacking [3]. 

New lasers are emerging, such as the nonablative fractional laser, for the treatment of scarring although evidence of efficacy is again largely anecdotal [49]. Pulsed-dye lasers may be useful in treating resistant keloids in combination with intralesional steroids [3, 50]. They may also flatten hypertrophic scars and reduce erythema although with conflicting reports of success [51]. So called “laser welding” of skin wounds seems to produce better scars in rats [52]. 

Cryotherapy tends to be limited to the treatment of very small scars because of the attendant side-effects of pigmentation changes, skin atrophy, and pain. However, a method of delivering intralesional cryotherapy using a needle attached to a liquid nitrogen source has been described in a small observational study which appears to be effective at shrinking keloid scars [53]. 

Radiotherapy in combination with surgery is an effective treatment of keloids but is limited in practice by the risk of carcinogenesis [38]. Other physical therapies include massage, ultrasound, medical tattooing/camouflage, static electricity, and pulsed electrical stimulation. These are as yet unproven by randomised controlled trials.

### 2.8. What Medical Antiscarring Therapies Exist?

Intralesional injection of corticosteroids, usually triamcinolone, is the most commonly accepted medical treatment of pathological scarring [3]. Steroids are most effective in the treatment of keloids rather than hypertrophic scars, particularly when combined with other modalities [54]. However, steroids do not improve normal scars and are marred by side effects such as depigmentation and telangiectasias, so many other potential medical therapies are under investigation. 

Interferon has been shown to increase collagen breakdown, improve hypertrophic scars, and prevent recurrence of keloids better than triamcinolone [3]. However, topical imiquimod (interferon *α* 2 inducer) and interferon *α*2b have both recently been found to be ineffective in the treatment of normal scars and keloids, respectively [55, 56].

The cytotoxic cancer chemotherapy drugs 5-fluorouracil and bleomycin have so far been used successfully to flatten hypertrophic and keloid scars in small studies [57, 58]. 

Other antiscarring therapies under investigation include onion extract, AZX100 (a phosphopeptide analogue of HSP20 [heat-shock-related protein]), pentoxifylline, prolyl-4 hydroxylase, verapamil, tacrolimus, and anti-TNF-*α* agents [59–65].

Perhaps the most promising potential medical therapy stems from the research into fetal scarring and TGF-*β*. In particular, an improvement in scarring in rat wounds has been shown by neutralizing TGF-*β*1 alone or both TGF-*β*1 and 2 (with antibodies or competitive inhibition with mannose-6-phosphate which inhibits TGF-*β* activation) or alternatively by adding TGF-*β*3 [26]. These findings have led to testing of new agents that are now undergoing phase II and phase III clinical trials [66, 67].

### 2.9. How Can We Improve Scars Surgically?

Meticulous surgical technique remains vitally important in the final scarring outcome. However, using tissue adhesive glue in place of sutures demonstrates better scars in randomised controlled trials in breast and head and neck surgery patients [68]. Paper tape shows equivalent cosmetic outcomes in the treatment of pediatric facial lacerations and reduces the incidence of hypertrophic scarring caesarean section scars when applied for 12 weeks [69, 70].

In revision surgery, the traditional methods of re-excision and Z- or W-plasties (reorientating scars), grafts or flaps followed by adjuvant therapy, remain the mainstay of treatment but newer techniques are now available such as dermabrasion, chemical peels, and follicular unit micrografting [71]. Other advances have focused on reducing wound tension. One method avoids tension by leaving the dermal element of the scar unexcised and closing epithelial skin flaps over the dermal scar. Thus the scar continues to take up the tension in the dermis but any widening is effectively hidden [72]. The similar “fillet flap” has been described for revising keloid scars. The skin over the keloids are raised as flaps, the keloid tissue excised, and the skin resutured thus closing the wound without tension [73]. Alternatively a split thickness skin graft can be applied to the wound bed. Tension is avoided and the graft naturally contracts over time [74]. 

Caution should be applied in the practise of these new techniques as their evidence base is largely anecdotal or based on small series.

## 3. Conclusion

Scarring research has seen advances in scar assessment, prevention, and revision. Many scar treatments are being trialled. A central, although not exclusive, role of transforming growth factor beta has emerged and the possible aetiologies of pathological scars are gradually being defined. The practise of surgery promises to be significantly improved in near future by the advent of effective antiscarring therapies and perhaps ultimately, completely scar-less healing. 

##  Summary Points

An effective therapy for the prevention/treatment of cutaneous scarring may have application in fibrosis of other tissues.

Scarring remains a significant adverse consequence of surgery, trauma, and burns with current therapies having poor efficacy and evidence base.

First-line therapy of hypertrophic scars is silicone gel/sheeting and first-line therapy for keloids is intralesional steroid injection.

Correct orientation of incisions parallel to the lines of Langer, reducing wound tension and applying paper tape can help to prevent postoperative hypertrophic scarring.

##  Tips for Nonspecialists

Normal scars take a year to mature and should be flat and pale. Red, raised, or painful scars are hypertrophic or even keloid and should be treated as such.

Symptomatic scars can be improved and patients with troublesome scars should be referred to a plastic surgeon.

##  Ongoing Research

Research into fetal (nonscarring) cutaneous healing and transforming growth factor-*β* are at the forefront of attempts to design an antiscarring drug.

Manipulation of the TGF-*β* isoforms and related bioactive molecules to inhibit scar production understanding why some scars become pathological.

A range of medical therapies are being trialled including cytotoxic drugs, interferon and onion extract and AZX100 (please note that this is not an exhaustive list).

Plastic surgical techniques to revise scars and treat pathological scars.

##  Unanswered Questions

Why do some tissues scar but not others?

How do steroids and silicone improve pathological scars?

How can we more accurately predict which patients may develop pathological scars following surgery or injury?

## Figures and Tables

**Table 1:** Normal, hypertrophic, and keloid scars compared.

Normal	Hypertrophic	Keloid
Confined to edges of original wound	Confined to edges of original wound	Extends beyond edges of wound
Gradual fading and atrophy after maturation	Regresses after initial peak (although often over several years)	Progressive
No treatment required	First-line treatment: silicone patches or gels	First-line treatment: intralesional steroid injection
Best in the elderly	Worst in the young	More common in darker skin
Normal response to TGF-*β*1	Abnormal response to TGF-*β*1	Failure of apoptosis
